# Evaluation of a Web-Based E-Learning Platform for Brief Motivational Interviewing by Nurses in Cardiovascular Care: A Pilot Study

**DOI:** 10.2196/jmir.6298

**Published:** 2016-08-18

**Authors:** Guillaume Fontaine, Sylvie Cossette, Sonia Heppell, Louise Boyer, Tanya Mailhot, Marie-Josée Simard, Jean-Francois Tanguay

**Affiliations:** ^1^ Montreal Heart Institute Research Center Montréal, QC Canada; ^2^ Faculty of Nursing Université de Montréal Montréal, QC Canada; ^3^ Montreal Heart Institute Montréal, QC Canada; ^4^ Center for Innovation in Nursing Education Montréal, QC Canada; ^5^ Faculty of Medicine Université de Montréal Montréal, QC Canada

**Keywords:** motivational interviewing, cardiovascular diseases, coronary artery disease, health behavior change, Web-based learning, e-learning, pilot study

## Abstract

**Background:**

Brief motivational interviewing (MI) can contribute to reductions in morbidity and mortality related to coronary artery disease, through health behavior change. Brief MI, unlike more intensive interventions, was proposed to meet the needs of clinicians with little spare time. While the provision of face-to-face brief MI training on a large scale is complicated, Web-based e-learning is promising because of the flexibility it offers.

**Objective:**

The primary objective of this pilot study was to examine the feasibility and acceptability of a Web-based e-learning platform for brief MI (MOTIV@CŒUR), which was evaluated by nurses in cardiovascular care. The secondary objective was to assess the preliminary effect of the training on nurses’ perceived brief MI skills and self-reported clinical use of brief MI.

**Methods:**

We conducted a single-group, pre-post pilot study involving nurses working in a coronary care unit to evaluate MOTIV@CŒUR, which is a Web-based e-learning platform for brief MI, consisting of two sessions lasting 30 and 20 minutes. MOTIV@CŒUR covers 4 real-life clinical situations through role-modeling videos showing nurse-client interactions. A brief introduction to MI is followed by role playing, during which a nurse practitioner evaluates clients’ motivation to change and intervenes according to the principles of brief MI. The clinical situations target smoking, medication adherence, physical activity, and diet. Nurses were asked to complete both Web-based training sessions asynchronously within 20 days, which allowed assessment of the feasibility of the intervention. Data regarding acceptability and preliminary effects (perceived skills in brief MI, and self-reported clinical use of conviction and confidence interventions) were self-assessed through Web-based questionnaires 30 days (±5 days) after the first session.

**Results:**

We enrolled 27 women and 4 men (mean age 37, SD 9 years) in March 2016. Of the 31 participants, 24 (77%, 95% CI 63%–91%) completed both sessions in ≤20 days. At 30 days, 28 of the 31 participants (90%) had completed at least one session. The training was rated as highly acceptable, with the highest scores observed for information quality (mean 6.26, SD 0.60; scale 0–7), perceived ease of use (mean 6.16, SD 0.78; scale 0–7), and system quality (mean 6.15, SD 0.58; scale 0–7). Posttraining scores for self-reported clinical use of confidence interventions were higher than pretraining scores (mean 34.72, SD 6.29 vs mean 31.48, SD 6.75, respectively; *P*=.03; scale 10–50). Other results were nonsignificant.

**Conclusions:**

Brief MI training using a Web-based e-learning platform including role-modeling videos is both feasible and acceptable according to cardiovascular care nurses. Further research is required to evaluate the e-learning platform in a randomized controlled trial.

**Trial Registration:**

International Standard Randomized Controlled Trial Number (ISRCTN): 16510888; http://www.isrctn.com/ISRCTN16510888 (Archived by WebCite at http://www.webcitation.org/6jf7dr7bx)

## Introduction

### Background

Coronary artery disease contributes significantly to worldwide morbidity and mortality [1]. According to clinical practice guidelines, the reduction of cardiovascular risk factors through health behavior change plays a critical role in treatment for coronary artery disease [2]. Smoking cessation, medication adherence, physical activity, and diet are often cited as examples of health behaviors that are amenable to change and allow risk factor reduction [3,4]. Health behavior change is determined mainly by *conviction* (ie, knowledge and understanding of the disease, personal meaning, and the relevance of that knowledge) and *confidence* in one’s ability to change successfully [5,6]. These determinants of health behavior change can be addressed via client-centered interventions that target individuals’ beliefs, values, and motivation [7-10].

Brief motivational interviewing (MI) is a client-centered approach designed to guide individuals through collaborative conversational style and to solidify their motivation and commitment to health behavior change [7,11]. Unlike longer interventions, brief MI was proposed to meet the needs of clinicians who have little time to use the full range of MI techniques in practice [12-14]. The scientific literature generally supports the efficacy of brief MI in various health care settings, including those involving smoking cessation, medication adherence, physical activity, and diet [7,14-17]. While brief MI is promising, health care practitioners often lack time, basic training, or continuous education opportunities to update their knowledge and skills regarding increasing clients’ motivation for change [18]. A systematic review [18] evaluated 10 studies involving health care practitioners’ use of brief to intensive MI training methods. The duration of the training ranged from 20 minutes to 24 hours, while the format varied from face-to-face sessions to short video modules presented in a classroom setting. Results of the review were generally favorable, suggesting that MI training generates an increase in knowledge, skills, and clinical use. In contrast, very few studies have thus far examined MI training delivered via Web-based e-learning. In fact, of 36 studies included in 3 different systematic reviews concerning MI training, none evaluated Web-based MI training [18-20].

E-learning, defined as instruction delivered on a digital device [21], has been shown to be effective for health care practitioners, with knowledge acquisition and clinical skill development equal or superior to those observed with face-to-face instruction [22-27]. Web-based e-learning can reduce the cost and time involved in providing continuing education, as it offers flexibility with respect to learning times and locations and can reach an unlimited number of clinicians [28]. Web-based e-learning can therefore enhance health care practitioners’ knowledge and skills, as a prerequisite for effective use of health behavior interventions such as brief MI [25,29].

User acceptance of Web-based e-learning for specific sociodemographic groups of health care practitioners, such as nurses, is a topic of great interest [30-32]. However, the literature concerning the subject is scarce. According to the unified theory of acceptance and use of technology, various factors influence user acceptance of technology, which in turn influences technology use [33]. Careful attention must then be paid to learners’ perceptions and attitudes toward workplace e-learning in order to optimize the knowledge, skills, and clinical use of brief MI [30,34]. The integration of interactivity measures and audiovisual media in e-learning may positively affect learners’ perceptions and attitudes [29].

Video-based e-learning showcasing clinical simulation has attracted strong interest from clinicians and researchers [23,35-37]. Videos can facilitate knowledge acquisition and clinical skill development through pedagogical material that matches the reality of clinical settings [36,38-40]. Video-based e-learning has the potential to “enliven abstract concepts, demonstrate real-world applications of complex principles, motivate the learner, organize thoughts and actions of highly cognitive processes, and heighten learner attention and interest” [36]. This is particularly interesting, because MI is usually learned through observation of role models in face-to-face or, most recently, videotaped clinical simulations [11,18].

However, little is known about the educational effectiveness of brief MI training via a Web-based e-learning platform. To our knowledge, cardiovascular nurses’ MI-related skill development and clinical use of brief MI have not been evaluated. Therefore, in this study, we developed and pilot tested a Web-based e-learning platform for brief MI, which included videos in which nurses could observe brief MI in a real-life clinical context.

### Study Objectives

The primary objective of this pilot study was to examine the feasibility and acceptability of a Web-based e-learning platform for brief MI (MOTIV@CŒUR), which was evaluated by nurses in cardiovascular care. The primary end point of the pilot study was the proportion of nurses who had completed both training sessions 20 days after the initiation of the training session.

The secondary objective was to assess the preliminary effect of MOTIV@CŒUR on nurses’ perceived skill in, and self-reported clinical use of, brief MI.

## Methods

### Study Design and Setting

We conducted a single-group, pre-post pilot study involving cardiovascular nurses to assess MOTIV@CŒUR. We conducted the study at the coronary care unit (CCU) at a tertiary care hospital center in Montreal, Canada. The pilot study was registered (ISRCTN16510888), as well as being approved by the Scientific and Ethics Committee of the Montreal Heart Institute Research Center (reference number: 2015-1948). Our study is reported in accordance with the CONSORT-EHEALTH checklist version 1.6.1 [41] (see Multimedia Appendix 1). No content or methodological modifications were made after study commencement.

### Participants

We recruited a convenience sample of nurses employed at the CCU. Nurses were eligible for participation if they were working at the CCU during the study period. The inclusion criteria were employment in a temporary replacement or permanent position at the CCU and basic computer skills. The exclusion criterion was completion of MI training in the preceding year.

### Procedure

Enrollment and follow-up occurred between March and May 2016 (see Table 1) [28,42-44]. We recruited nurses through individual face-to-face encounters at the CCU. Participants were informed that they would need to complete the training and study requirements on their personal time without financial compensation. However, it was stated that they would receive a certificate attesting to 1 hour of continuing education after completing the training. After receiving an explanation regarding the study and providing written consent, participants completed a paper-based sociodemographic questionnaire. An individual identification number, username, and password were then provided to participants, to allow them to log in to the e-learning platform throughout the study. They also received a training information sheet that explained MOTIV@CŒUR using screen captures and colorful textual content. During the 15-day period following enrollment, an initial email containing the URL for the Web-based e-learning platform was sent to each participant.

**Table 1:** Schedule of enrollment, interventions, and assessments for MOTIV@CŒUR.^a^

Participant timeline			Study period and time points				
			Enrollment	Experimentation			Closeout
			t_0_ days –20 to 0	t_1a_ day 1	t_1b_ day 1	t_2_ day 15 (±5 days)	t_3_ day 30 (±5 days)
**Enrollment**						
	Eligibility screen and informed consent		×				
**Intervention encounters**							
	Training sessions in brief MI				Session 1 (30 min)	Session 2 (20 min)	
**Assessments**							
	Sociodemographic questionnaire		×				
	**Primary objectives**						
		*Feasibility* of the Web-based e-learning platform for brief MI	×	×	×	×	×
		*Acceptability* of the Web-based e-learning platform for brief MI					×
	**Secondary objectives**						
		*Perceived skill in brief MI*		×			×
		*Self-reported clinical use of brief MI*		×			×

^a^Template adapted from the Standard Protocol Items: Recommendations for Interventional Trials (SPIRIT) guidelines [42].

^b^MI: motivational interviewing.

^c^Measured throughout the study with indicators from Feeley and Cossette [43].

^d^Measured with Cheng’s tool [28].

^e^Measured with the adapted tool of Paradis et al [44].

### The Web-Based E-Learning Platform for Brief MI: MOTIV@CŒUR

MOTIV@CŒUR (in French, which translates as MOTIV@HEART in English) is a Web-based e-learning platform for brief MI, which includes role-modeling videos. The intervention content is based on the work of key authors in brief MI [11,44-47].

#### Development Process

**Figure 1:**
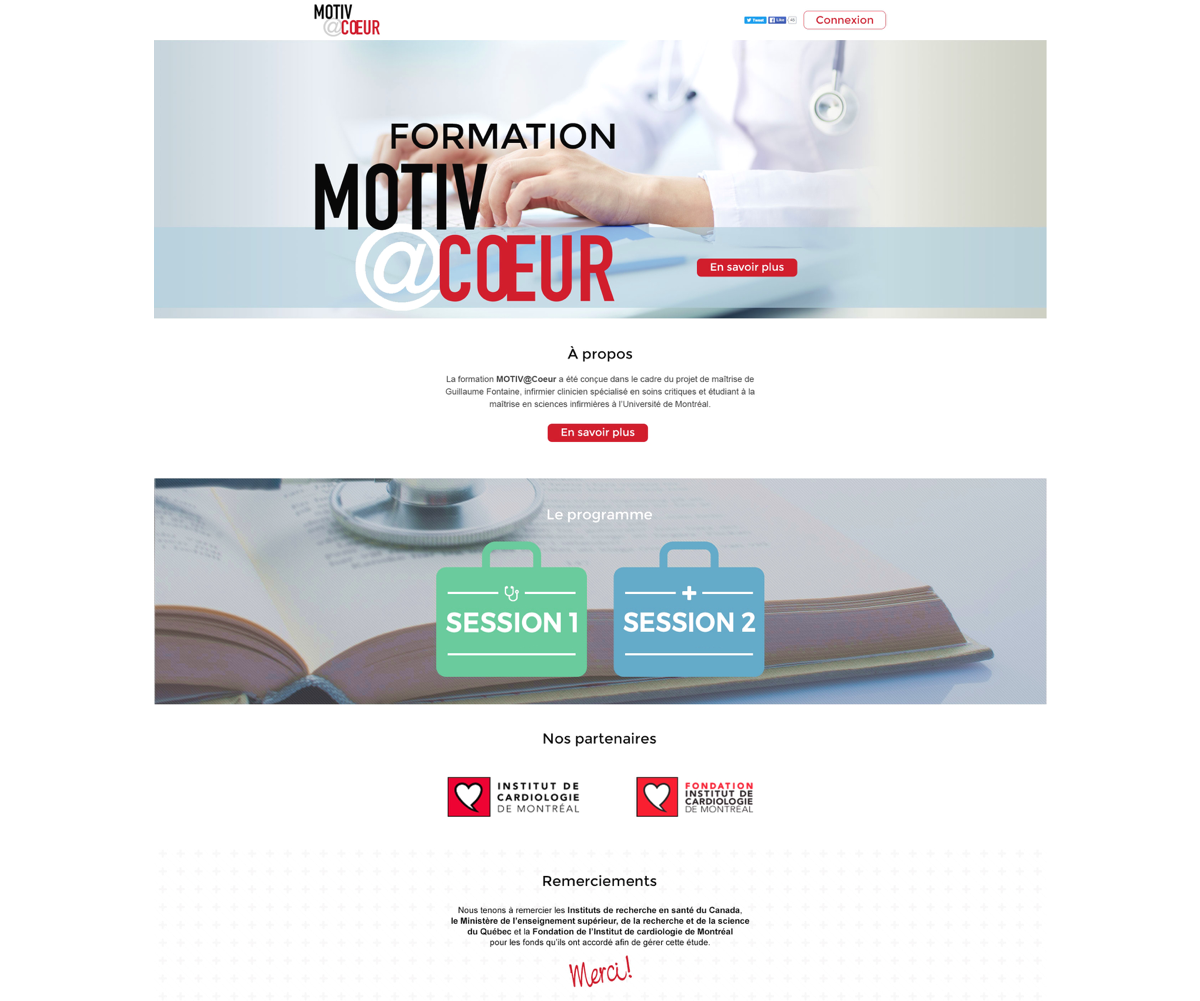
MOTIV@COEUR homepage (in French).

The MOTIV@CŒUR Web-based platform was developed by an independent consulting firm in Montreal, Canada. We chose the firm because it designs interactive websites whose format is adaptive to computers, tablets, and smartphones. MOTIV@CŒUR is based on the open-source learning platform Moodle 3.0 (Moodle Pty Ltd, Perth, Australia). The MOTIV@CŒUR homepage (see Figure 1) was designed to create an appealing first impression, using visual material and dynamic components.

Subsequent adaptation of the content of the e-learning platform for brief MI is possible via access to the Moodle course management system provided to the research team. However, changes in the design of the website require the involvement of the consulting firm.

To ensure the preservation of data related to the implementation MOTIV@CŒUR and the usage statistics for nurses, the website and data regarding its use were hosted on secure computer servers at the research setting for the duration of the study.

#### MOTIV@CŒUR Access

MOTIV@CŒUR can be accessed via a fixed URL. The availability of the website was restricted to the study period. Participants could log in to the e-learning platform from home or work via the device of their choice, using their personal log-in credentials, which were provided during the face-to-face encounter at the CCU. We suggested that participants change their passwords after the initial log-in. Passwords could also be reset via their personal email accounts if forgotten.

#### MOTIV@CŒUR Content

The content of the brief MI on the Web-based e-learning platform was developed by the project team, which included 1 MI expert and an experienced cardiology researcher, who supervised the development of the intervention, led by GF. In addition, 2 MI experts validated the content. The intervention was pretested with 5 nurses who were not part of the sample of nurse participants in this study. We adjusted MOTIV@CŒUR content according to the nurses’ comments before we recruited the study sample.

**Figure 2:**
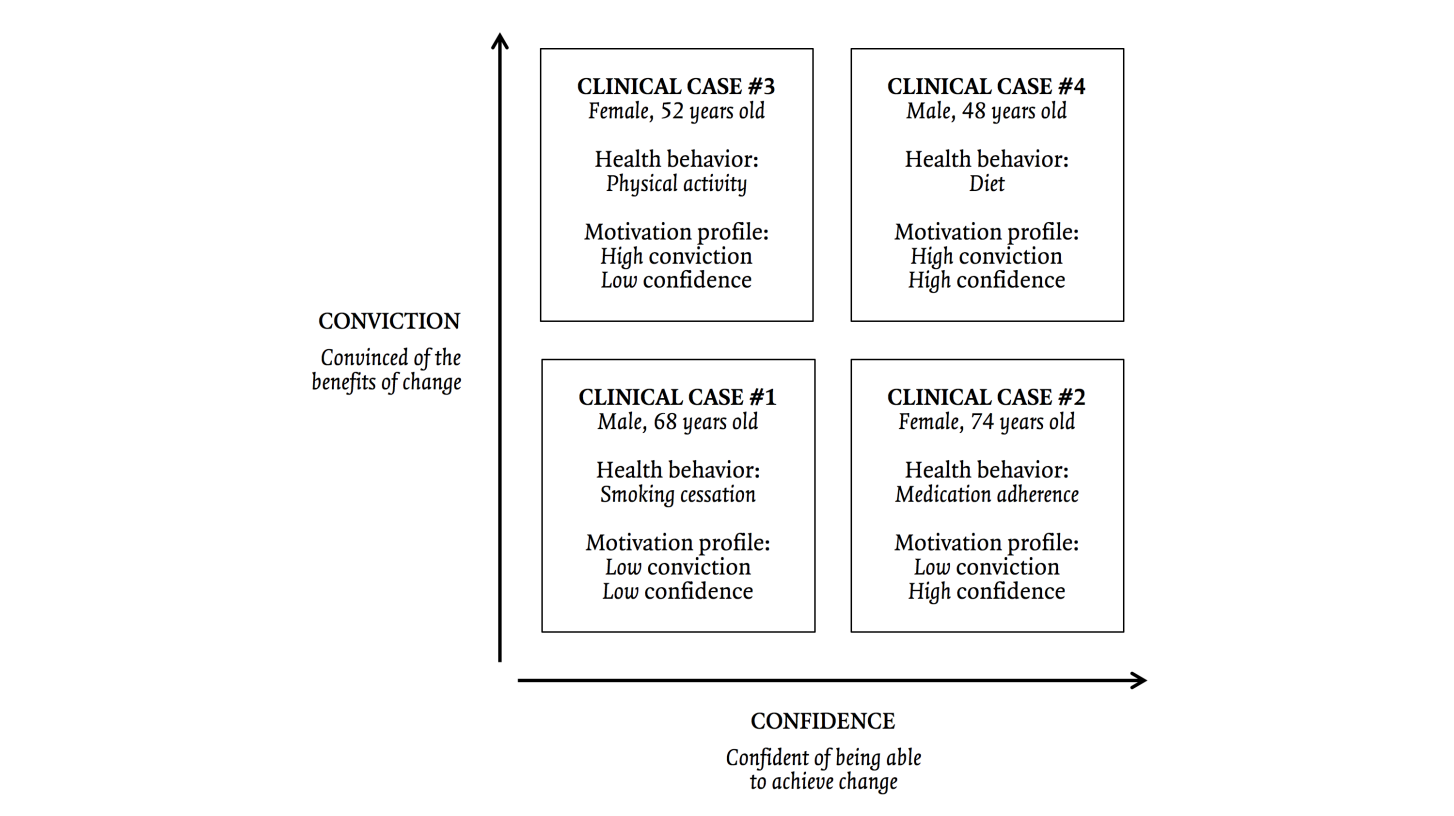
The 4 clinical cases and motivation profiles presented in MOTIV@CŒUR.

MOTIV@CŒUR was designed around 4 scenarios, each presenting a clinical case involving a client with a given level of conviction (low or high) and confidence (low or high) regarding change in a health behavior (see Figure 2) [47]. Each motivation profile was associated with one of the following health behaviors: smoking cessation, medication adherence, physical activity, or diet. For instance, clinical case #1 presents the association between low conviction and confidence levels for smoking cessation. We chose 4 different associations between motivation levels and health behaviors as examples that could be extrapolated to other health behaviors for individuals with any motivation profile. The team developed scenarios for each clinical case, based on real-life experience. During the scenarios, the nurse introduced herself, targeted the health behavior in each clinical case, assessed the level of conviction and confidence regarding change, and engaged the client in a brief MI conversation. Following each scenario, a second video showed the cardiology nurse practitioner (CNP) explaining why each intervention was retained in response to the client’s motivation profile.

The content of brief MI for the 4 scenarios was based on the model developed by Bédard [47], who adapted the work of Miller and Rollnick [11]. In this model, *conviction* represents the extent to which each individual perceives practical and emotional benefits to the change of a health behavior. *Confidence* represents the extent to which the individual is confident of being able to achieve change [47]. After assessing the client’s motivation, the practitioner provides tailored brief MI to increase conviction and confidence regarding health behavior change. Through videotaped role modeling, nurses could observe the CNP involving brief MI in a real-life clinical context. Videos were recorded at the research setting in a real patient room, to represent the real-life context (see Multimedia Appendix 2 for screenshots), with 4 volunteers (2 men and 2 women) representing different ages.

MOTIV@CŒUR was conceptualized to ensure that participants would observe real-life examples, allowing them to (1) familiarize themselves with the spirit of brief MI, (2) acquire basic skills in brief MI (eg, open-ended questions, validation, and reformulation), (3) recognize and reinforce the change discourse, specifically that involving conviction and confidence, (4) learn to create and strengthen the change discourse, (5) learn to accept resistance to avoid confrontation, (6) understand how to develop a plan, and (7) understand how to help clients to initiate change [11,48].

#### MOTIV@CŒUR Structure

**Figure 3:**
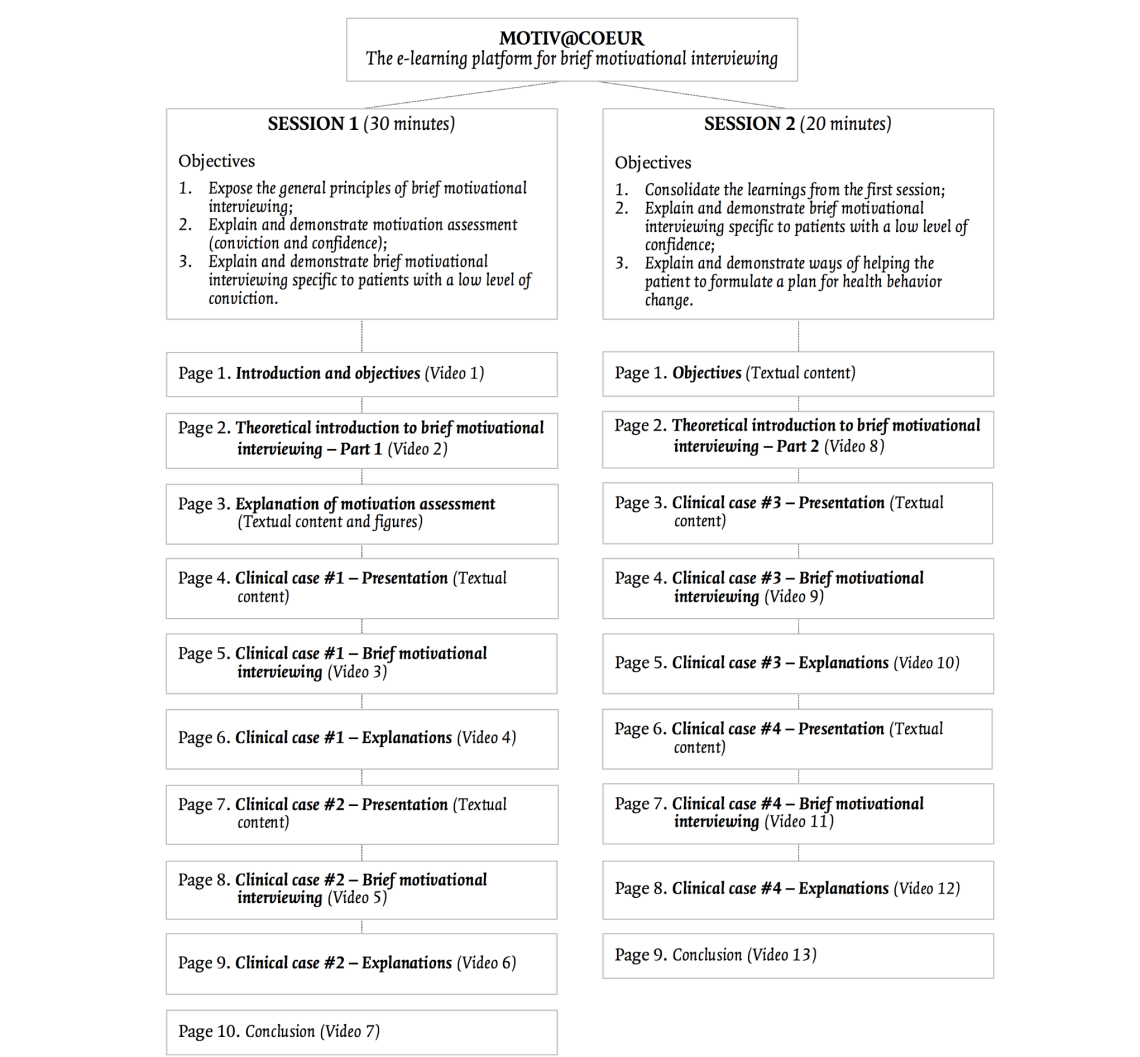
Structure of MOTIV@CŒUR, a web-based e-learning platform for brief motivational interviewing.

MOTIV@CŒUR consists of 2 training sessions including 13 video modules (see Figure 3). The planned durations were 30 minutes for the first session (S1) and 20 minutes for the second session (S2). Following an introduction and statement of objectives, each session was initiated with a video containing a theoretical introduction to brief MI. Each clinical case was then separated into 3 sections: (1) a textual presentation of the clinical case on the screen, (2) a video of brief MI, in which the CNP interacted with each client, and (3) a video in which the CNP explained why each intervention was retained in response to the client’s motivation profile. Both sessions concluded with a reminder of the key concepts and tips for real-world use of brief MI.

#### Use Parameters

We asked participants to complete S1 within 5 days of receiving the initial email sent after enrollment. Two options were provided for completing the first MOTIV@CŒUR Web-based training session. Participants could complete the Web-based training individually in a dedicated room equipped with computers at the study hospital, during a scheduled session in which a facilitator would explain the project and procedure for accessing MOTIV@CŒUR to each participant. Participants could also complete the training at home with remote support (eg, by email or telephone). In addition, they were encouraged to practice brief MI techniques observed in the video in their regular clinical practice, if appropriate.

We asked nurses to complete S2 either at home or at the hospital, 2 weeks after S1. There was no computer constraint limiting completion of S2 earlier or later than this. However, participants were required to complete both sessions within 20 days.

#### Reminders, Level of Human Involvement, and Co-interventions

We planned a maximum of 3 email or telephone reminders at 3-day intervals for each of the 3 time points in the study (S1, S2, and outcome measures). A maximum of 9 emails or telephone reminders could be sent throughout the study period.

The intervention was completely asynchronous. The research team was available at all times, to provide technical support in person or via mail or telephone. Apart from the technical support provided when necessary (access to the website, log-in, and password), we offered no other intervention, such as that involving information and content explanation regarding the brief MI.

### Outcome Measures

The primary feasibility outcome was the completion of both training sessions 20 days after initiation of S1. We also assessed additional feasibility outcomes regarding recruitment and study completion.

Secondary outcomes included the acceptability of MOTIV@CŒUR according to the cardiovascular nurses, skills perceived in brief MI, and self-reported clinical use of brief MI with coronary patients. These outcomes were self-assessed via Web-based questionnaires.

#### MOTIV@CŒUR Feasibility

We measured the feasibility of the Web-based e-learning platform for brief MI from recruitment to closeout using indicators collected throughout the study period, based on pilot study evaluation criteria established by Feeley and Cossette [43].

Feasibility indicators collected by the research team included the proportion of enrolled nurses in the eligible population, recruitment duration, and completion of outcome measures.

Feasibility indicators were also extracted from the Moodle platform. Moodle collects information about each user with an exact time stamp for each action (ie, change of a page in a module or completion of a module). We interpreted the interval between 2 actions as engagement with the site or absence from the site. Since an inappropriately long latency period between the user’s actions would overestimate the time spent on each session, we defined a maximum latency period, fixed at 15 minutes. When a latency period exceeded this threshold, we deducted it from the time spent on MOTIV@CŒUR. Feasibility indicators extracted from the Moodle platform for each user included the duration of each session, number of sessions completed, and time elapsed between the completion of S1 and S2.

We also recorded types, numbers, and timestamps for reminders sent to participants in an Excel file, version 15.16 (Microsoft).

#### MOTIV@CŒUR Acceptability

We used the model of information systems quality antecedents on nurses’ acceptance of e-learning, developed by Cheng [28], to assess posttraining acceptability of the Web-based e-learning platform for brief MI. The tool evaluates nurses’ perception of the e-learning system, using 27 items grouped into 2 main dimensions: global system quality and technology acceptance. These dimensions are based on DeLone and McLean’s [49] work in information systems quality and van der Heijden’s [50] technology acceptance model. The model is subdivided into 8 subdimensions, of which 4 are related to global system quality (system, information, service, and user interface design quality), and 4 are related to technology acceptance (perceived usefulness, perceived ease of use, perceived enjoyment, and intention to use). The items in each subdimension were described the original paper [28]. Responses are provided using a Likert scale ranging from 1 (strongly disagree) to 7 (strongly agree), with 4 representing neutral responses. The score for each subdimension is calculated by summing the scores for the responses to the items therein and dividing the result by the number of items in the subdimension. A higher total score indicates greater acceptability (possible range: 0–7). Cronbach alphas for the scale were between .70 and .96 in previous studies [28]. The tool was translated into French using the back-translation method defined by the World Health Organization [51]. The content validity of the translated items was then determined by an expert, who provided adjustments to the language and structure of the items. A pretest was performed and included nurses who were not involved in the project.

Nurses could also provide suggestions and comments regarding MOTIV@CŒUR at the end of the acceptability questionnaire.

#### Preliminary Efficacy of MOTIV@CŒUR

We adapted the tool of nursing interventions specific to conviction and confidence levels and stages of change developed by Paradis et al [44], to assess perceived skill in brief MI and the self-reported clinical use of brief MI before and after training. We reduced the number of interventions from 55 to 26, retaining only those that targeted conviction and confidence, as this was the primary focus of the brief MI training in the study. The content was then validated by 2 MI experts.

The scale contained 26 intervention items grouped under 2 motivational intervention dimensions: conviction (16 items) and confidence (10 items). Based on the work of Cossette et al [52], 2 questions were asked for each intervention item to assess outcomes. The first question (“How comfortable do you feel doing it?”) assessed nurses’ perceived skill in performing each intervention. The second question (“How often do you do it?”) assessed nurses’ self-reported clinical use of each intervention. Each question was used to calculate a total score and 2 subdimension scores for conviction and confidence. The response scale for each question ranged from 1 (not at all) to 5 (extremely) and provided 2 total scores ranging from 26 to 130. A higher score for the first question indicated higher perceived skill in brief MI, and a higher score for the second question indicated higher clinical use of brief MI. To calculate total scores, we recoded a maximum of 3 missing values per participant in the mode for each item.

#### Other Measures

A self-administered sociodemographic paper questionnaire was completed at enrollment to collect data regarding nurses’ general profiles concerning sex, age, language, educational level, year of entry to the hospital, employment status, duration of experience in nursing and cardiovascular acute care, shift, and type of position held at the CCU. We also asked participants whether they had previously completed Web-based training.

### Sample Size

To examine the primary feasibility outcome, we defined success as the completion of both training sessions by 80% of participants within 20 days. We expected this rate to be 80%; therefore, we targeted a sample of 30 participants to allow estimation with accuracy of ±14.3% and a confidence level of 95%.

### Statistical Analysis

With respect to sociodemographic, acceptability, and preliminary effect variables, we calculated means and SDs for continuous variables, and counts and percentages for categorical variables. We also used descriptive statistics for the feasibility criteria, as follows: (1) proportion of enrolled nurses in the eligible target population (expected: 50%), (2) time required to complete recruitment (expected: 60 ± 30 days), (3) proportion of nurses who completed both sessions in ≤20 days (expected: 80%), (4) proportion of nurses who completed both sessions in 60 ± 10 min (expected: 80%), and (5) proportion of nurses who completed outcome measures (expected: 80%).

We compared changes between pre- and posttraining measures for perceived skill in brief MI and self-reported clinical use of brief MI using Student *t* test for paired samples, with a 2-sided significance level of .05. We also performed Student *t* test for paired samples, with the same parameters used, for the 4 subdimension scores for pre- and posttraining conviction and confidence. All statistical tests were 2-sided and performed using IBM SPSS version 23.0 (IBM Corporation). We verified basic assumptions, such as normal distribution, before analysis.

## Results

### Participant Characteristics

Most participants were women, and participants’ mean age was 37 years (see Table 2). The majority had completed university-level education and worked full-time as bedside nurses. The duration of participants’ experience as critical cardiovascular care nurses ranged from 1 month to 37 years, with a mean of 11 years. Nurses were almost evenly distributed across all work shifts, with 5 working rotating shifts. More than three-quarters of participants had previously completed Web-based training, but none had undertaken MI in the preceding year.

**Table 2:** Nurses’ baseline sociodemographic data (N=31).

Characteristic		Mean (SD) or n (%)
Sex (female), n (%)		27 (87%)
Age, in years, mean (SD)		37 (9)
Education (Bachelor’s degree or higher), n (%)		18 (58%)
Position (full-time), n (%)		18 (58%)
Position in coronary care unit (bedside nurse), n (%)		27 (87%)
Experience in acute care, in years, mean (SD)		11 (10)
**Shift, n (%)**		
	Day	9 (29%)
	Evening	9 (29%)
	Night	8 (26%)
	Rotation	5 (16%)
Previously completed Web-based training (yes)		24 (89%)^a^

^a^n=27.

**Figure 4:**
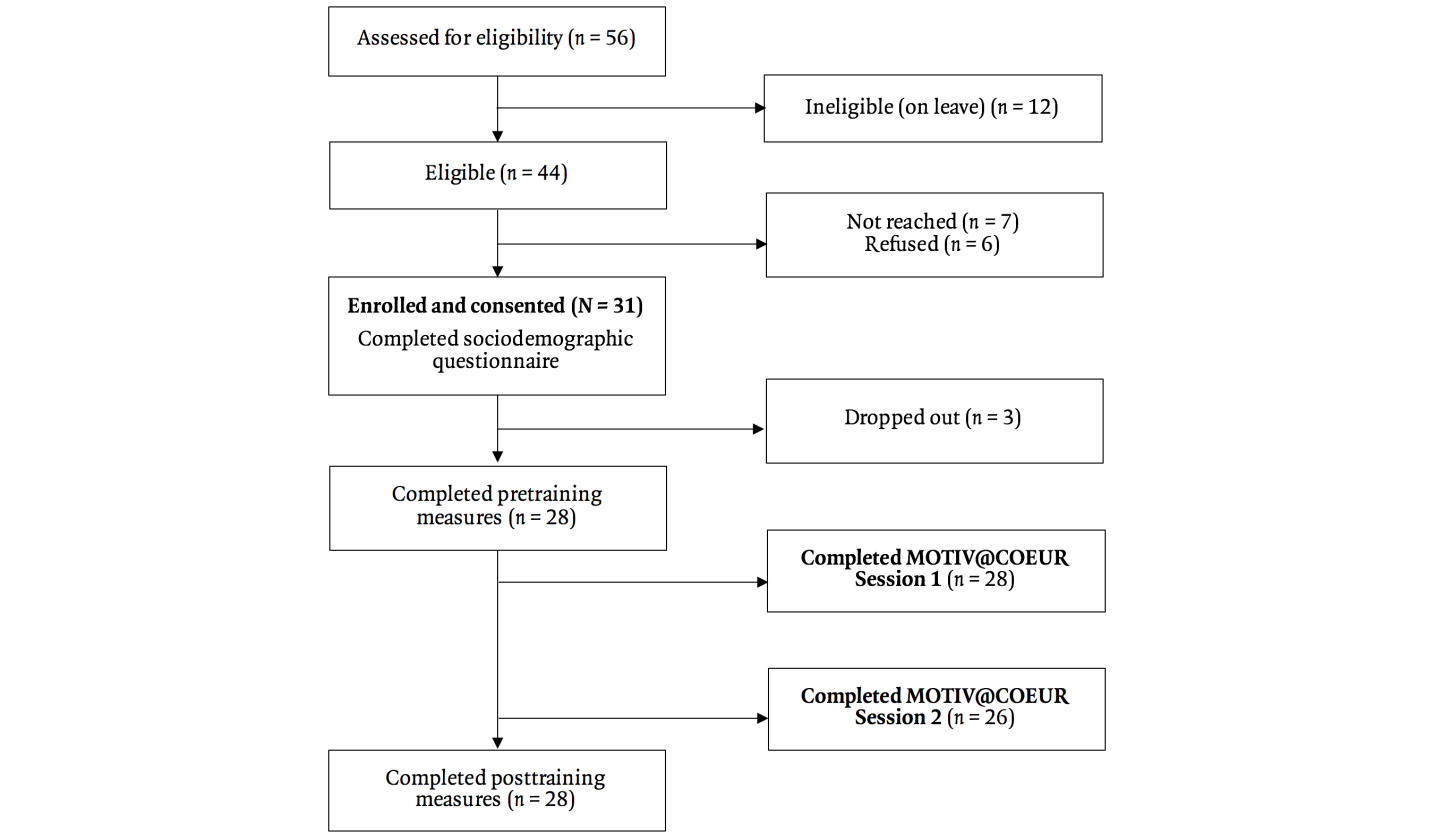
MOTIV@CŒUR study participation flowchart.

### Feasibility Results

#### Recruitment

The feasibility criteria for participant recruitment were all met. Of the 56 nurses employed at the CCU, 44 were eligible for study participation, and 31 (70%) were enrolled in the study between March and May 2016 (see Figure 4). This exceeded the target proportion of 50%. Moreover, recruitment was completed within 11 days, which was a significantly shorter period than the expected period of 30–90 days.

#### Training Nurses via the Web-Based E-Learning Platform for Brief MI

With regard to the primary feasibility outcome, 24 of the 31 recruited participants (77%, 95% CI 63%–92%) completed both training sessions within 20 days following initiation of S1 (see Table 3). This was close to the criterion for determining success (ie, 80%). Another 2 nurses completed S2 within 26 and 30 days of S1. In addition, 28 participants had completed S1 and 26 had completed S2 at 30 days. A total of 3 participants dropped out before beginning the training, resulting in 28 participants completing pretraining and posttraining measures.

**Table 3:** Feasibility of MOTIV@CŒUR (N=31).

Feasibility or outcome variable			No. or n (%)
**Feasibility criteria**			
	1. Nurses in the eligible target population (expected: 50%), n (%)		31 (70%)
	2. Duration of recruitment (expected: 30–90 days), no. of days		11
	3. Completed both sessions within ≤20 days (expected: 80%), n (%)		24 (77%)
		Completed the first session at 30 days, n (%)	28 (90%)
		Completed both sessions at 30 days, n (%)	26 (84%)
	4. Completed both sessions within 60 ± 10 min (expected: 80%), n (%)^a^		19 (73%)
	5. Completed posttraining measures (expected: 80%), n (%)		28 (90%)
**Completion of pretraining measures and first session, n (%)**			
	Completed before a reminder was sent		10 (32%)
	Completed after 1 reminder was sent		10 (32%)
	Completed after 2 reminders were sent		7 (23%)
	Completed after 3 reminders were sent		1 (3%)
	Never completed		2 (6%)
**Completion of second session, n (%)** ^b^			
	Completed before a reminder was sent		14 (50%)
	Completed after 1 reminder was sent		10 (36%)
	Completed after 2 reminders were sent		0
	Completed after 3 reminders were sent		2 (7%)
	Never completed		2 (7%)
**Completion of posttraining measures, n (%)** ^b^			
	Completed before a reminder was sent		13 (46%)
	Completed after 1 reminder was sent		8 (29%)
	Completed after 2 reminders were sent		6 (21%)
	Completed after 3 reminders were sent		1 (4%)
	Never completed		0

^a^n=26.

^b^n=28.

The results showed that 25 participants completed S1 and 22 completed S2 during a single connection. The mean durations were 31 (SD 6) minutes for S1 and 19 (SD 6) minutes for S2. The mean total training duration was 50 (SD 11) minutes, which was consistent with the expected duration for MOTIV@CŒUR. The mean period between the completion of S1 and S2 was 13 (SD 7) days, which was close to the recommended time of 2 weeks.

Of the 31 participants, 10 (32%) completed the baseline measures and S1 without requiring a reminder after the initial email providing instructions regarding accessing the Web-based e-learning platform. This proportion was higher in the rest of the study: 14 of 28 participants (50%) completed S2 without a reminder, and 13 of 28 participants (46%) completed the outcome measures without a reminder. Across the 3 time points, the first email reminder was more effective than the second and third reminders and doubled the number of participants who fulfilled the requirements.

In total, 80 emails and telephone reminders were sent throughout the study period. More specifically, 44 email, 16 telephone, and 20 voicemail reminders were sent. Of these, the email reminders were the most effective. Of the 44 email reminders, 27 (61%) resulted in the completion of requirements at each time point (S1, S2, and outcome measures), while 9 of 16 (56%) telephone reminders and 9 of 20 (45%) voicemail reminders were effective throughout the study period.

### Acceptability Outcomes

The Web-based e-learning platform for brief MI was considered highly acceptable by cardiovascular nurses across all 8 dimensions of Cheng’s [28] model (see Table 4).

**Table 4:** Posttraining acceptability of MOTIV@CŒUR (n=28).

Outcome variable		No. of items	Possible range	Mean (SD) score
**Global system quality**		15	0–7	5.95 (0.48)
	System quality	5	0–7	6.15 (0.58)
	Information quality	4	0–7	6.26 (0.60)
	Service quality	3	0–7	5.28 (0.96)
	User interface design quality	3	0–7	6.12 (0.69)
**Technology acceptance**		12	0–7	5.90 (0.75)
	Perceived usefulness	3	0–7	5.64 (0.81)
	Perceived ease of use	3	0–7	6.16 (0.78)
	Perceived enjoyment	3	0–7	5.80 (1.01)
	Intention to use	3	0–7	6.01 (0.84)

The 4 dimensions concerning system quality were evaluated favorably, and each received a mean score of >5 on the 7-point Likert scale. The 3 items that received the highest scores for system quality were the flexibility of MOTIV@CŒUR regarding learning time and location, presentation of course materials in a readable multimedia format, and the delivery schedule for the learning content. The 3 items that received the lowest scores were related to the quality of support services, as 11 nurses did not use them and provided neutral scores, which were below the observed scores of >5 for other items. The information quality subdimension received the highest score in the dimension related to global system quality.

The 4 dimensions concerning technology acceptance were evaluated very favorably by all participants and received scores of >5 on the 7-point Likert scale. The 3 items that received the highest scores were related to the ease of use of MOTIV@CŒUR, the usefulness of MOTIV@CŒUR for learning, and the opinion that MOTIV@CŒUR should be available to other nurses and professionals. While most participants agreed or strongly agreed that MOTIV@CŒUR was useful in their learning, they appeared less convinced of the superiority of e-learning relative to traditional face-to-face methods. Indeed, the 3 items that received the lowest scores but still scored >5 were related to enhanced learning effectiveness compared with other training methods, increased learning efficiency, and enjoyment while training with MOTIV@CŒUR. Finally, the overwhelming majority of participants agreed or strongly agreed that they would use the e-learning platform again if it were made available with more content and resources.

Comments of participants at the end of the acceptability questionnaire underlined the simplicity, clarity, and dynamism of the e-learning platform. One participant suggested developing a checklist on the training content to be made available to nurses in the clinical setting. Another participant proposed conducting practical workshops to implement the learning acquired during the Web-based training. Overall, the feedback from participants was positive and indicated significant interest in the Web-based e-learning platform for brief MI.

### Preliminary Efficacy Outcomes

Regarding the preliminary efficacy of MOTIV@CŒUR with respect to perceived skill in brief MI, posttraining scores for all dimensions were higher than pretraining scores. However, the raw differences were small and nonsignificant (see Table 5).

**Table 5:** Preliminary effect of MOTIV@CŒUR on perceived skill in brief motivational interviewing (MI) and self-reported clinical use of brief MI.

Outcome variable		No. of items	Possible range	Mean (SD) scores		*P* value
				Pretraining	Posttraining	
**Perceived skill in brief MI** ^a,b^		26	26–130	95.19 (16.37)	97.50 (15.38)	.40
	Conviction interventions	16	16–80	60.62 (9.79)	61.53 (8.87)	.54
	Confidence interventions	10	10–50	34.59 (7.01)	35.93 (6.98)	.30
**Self-reported clinical use of brief MI** ^a,c^		26	26–130	89.60 (15.74)	94.28 (13.64)	.13
	Conviction interventions	16	16–80	58.12 (9.44)	59.56 (7.97)	.41
	Confidence interventions	10	10–50	31.48 (6.75)	34.72 (6.29)	.03

^a^Higher is better.

^b^n=26.

^c^n=25.

In addition, regarding the preliminary efficacy of MOTIV@CŒUR with respect to self-reported clinical use of brief MI, posttraining scores for all dimensions were higher than pretraining scores. A significant effect was observed for self-reported clinical use of brief MI to increase clients’ confidence in change (*P*=.03). Other results were nonsignificant.

## Discussion

This study involved the design, implementation, and evaluation of a Web-based e-learning platform for brief MI, which included role-modeling videos for nurses in cardiovascular care. We demonstrated the feasibility, acceptability, and preliminary efficacy of the intervention. In addition, preliminary posttraining results regarding perceived skill and clinical use of brief MI were all more favorable than those observed in the pretraining assessment. Overall, the feedback received from participants was positive.

While some previous studies examined Web-based MI training with health care practitioners [53-56], to our knowledge, this study was the first to examine cardiovascular nurses’ evaluation of an asynchronous Web-based e-learning platform for brief MI. We were successful in recruiting 31 participants within 11 days, of whom 28 completed posttraining measures. This demonstrates cardiovascular care nurses’ significant interest in Web-based e-learning and interventions targeting health behavior change. The strong participation in the study could reflect the applicability and credibility of the use of brief MI in acute care settings. Brief MI demonstrated in the MOTIV@CŒUR videos lasted 3–4 minutes. This duration is more likely to be feasible in clinical settings than in longer motivational interventions [12-14].

Previous studies suggested that technical difficulties, such as a lack of Internet access, could impede the ease with which information and communication technology could be used by health care practitioners [22,57-59]; however, this was not the case in our study, as we did not experience problems with computers. We informed nurses that they were required to be at ease with basic computer use, prior to enrollment, and the research team was available for prompt technical support via email. The e-learning training progressed very well without significant technical difficulties. Participants asked occasional questions (eg, regarding a malfunctioning URL link), but no one experienced difficulty in using the Web-based e-learning platform. This could suggest that nurses in acute care settings are familiar with the use of information and communication technology for clinical and pedagogical purposes. Of the 27 participants who completed the acceptability measures in this study, 24 (89%) had previously completed Web-based training for other topics. The streamlining and improvement of the user interface design in Web-based training platforms could also have affected the ease with which participants used the system [60].

Participant reminders are often overlooked but crucial to asynchronous Web-based e-learning. Literature concerning the subject is scarce; only a few studies have been conducted, and they reported incomplete data regarding frequency, content, numbers, and mode of delivery (eg, telephone or email) for reminders sent to participants. For example, one study [61] proposed up to 3 automated email reminders for incomplete modules. Other studies included 2 automated emails sent 7 days apart, with an additional personalized email and telephone call if required [62], weekly reminders [63], and 2 reminders after 2 weeks [64]. This heterogeneity shows a lack of consensus regarding best practice with respect to the reminders sent to participants. In this study, we decided to send a maximum of 3 telephone or email reminders 3 days apart, at each time point to avoid oversoliciting participants. Two reminders ensured that approximately 90% of participants completed the sessions and measures. Relative to telephone and voicemail reminders, email reminders were more effective in ensuring the completion of requirements at each time point. This finding could inform future studies.

Our study’s high acceptability scores suggested that the Web-based e-learning platform for brief MI, based on Moodle, could be ready for inclusion in a larger study. However, some participants asked for further details and interactivity measures, which could be included in future iterations of the platform. The positive aspects of e-learning observed in this study, such as flexibility and control regarding the learning time and location, are consistent with those reported in the literature [22,25,26]. This could be explained by the adaptive format of MOTIV@CŒUR, which can be used anywhere via smartphones, tablets, and computers; however, we did not collect this information. Moreover, participants appreciated the presentation of MOTIV@CŒUR course materials in a multimedia format, as they all reported acceptability scores of >6 for this item in the posttraining assessment. This extends existing literature concerning the feasibility and acceptability of illustrating complex clinical processes, such as brief MI, in video modules [18,38,56].

The next step of this project is to optimize the tailoring, structure, and content of brief MI in the Web-based e-learning platform. Moreover, we intend to evaluate this platform in a randomized controlled trial, to assess its efficacy in comparison with alternative instructional methods such as face-to-face training and reading. Assessment of participant knowledge based on training content is an outcome we will explore in our future research. Moreover, objective measures are required for clinical skills and motivational interventions provided in health care settings. We also intend to assess the effect of brief MI, provided by health care practitioners, on health behavior change in coronary clients.

Future research should assess tailored, interactive, Web-based e-learning platforms for brief MI, as this was not the focus of our study, and the scientific literature has demonstrated the efficacy of such features [29]. In addition to tailoring the platform to health care practitioners’ knowledge and experience, researchers should develop an algorithm that accounts for each participant’s characteristics and specific needs (for instance, some participants asked for additional content, while others were satisfied with what was provided in MOTIV@CŒUR). In doing so, they could ensure that every participant follows an individualized path that could lead to enhanced knowledge and clinical skills [25,29]. The efficacy of interactivity measures in e-learning has been demonstrated in the scientific literature [25,29]. Web-based e-learning platforms for brief MI could benefit from the inclusion of self-assessment questions, interactive models and figures, and thought-stimulating activities [29]. When combined with videos, these elements are potentially valuable for scientific, pedagogical, and clinical purposes.

Future research should explore how to assess the effects of Web-based e-learning for brief MI on objective results in clinical settings. Indeed, despite the progress that has been made in recent years, evaluating the effects of e-learning on real clinical behavior and client outcomes remains a challenge [60,65]. With regard to clinical behavior, researchers should assess the effect of new skills acquired via Web-based e-learning for brief MI on practice using methods other than those involving self-report measures. Supervised clinical simulations of brief MI in parallel with Web-based training could be an interesting means of assessing changes in clinical practice.

Regarding the clinical implications of the study, the results regarding feasibility and acceptability were encouraging and showed that cardiovascular nurses were willing and able to use a Web-based e-learning platform for brief MI to develop skills related to health behavior change. This suggests that Web-based training covering a larger scope of clinical situations and levels of motivation could be designed to assist health care practitioners in providing health behavior change interventions. These interventions could target a larger spectrum of risk factors other than those related to coronary artery disease.

### Strengths and Limitations of the Study

The strengths of the study include adherence to the study protocol, the prospective registration of the study, and encouraging feasibility and acceptability results. In addition, no MOTIV@CŒUR-related technical problems occurred during the study period.

The study demonstrated the potential of Web-based e-learning training for brief MI, but it was subject to some limitations. First, as it was a pilot study, it was not designed for adequate power. Second, the Web-based, self-administrated questionnaires used in the study are not objective measures of real clinical use of brief MI. Third, the single-group, pre-post study design did not allow for causal inferences.

Most participants had experimented with Web-based training prior to entering the study. This could provide a partial explanation as to why the Web-based e-learning platform showed such high levels of acceptability. A study with a more diverse population of nurses and other health care practitioners could be interesting and allow researchers to determine whether sociodemographic variables increase acceptability scores and affect knowledge acquisition and clinical outcomes. However, this proved difficult in this pilot study, as the small sample size did not allow for enough power.

Not all participants enrolled in the study ultimately used MOTIV@CŒUR for training in brief MI, as 3 individuals dropped out before beginning the training. However, the global participation rate in the study was superior to those observed in similar studies. Indeed, 28 of the 31 participants (90%) used MOTIV@CŒUR, and this proportion ranged from 82% to 89% in other studies [66-69]. While our study included cardiovascular nurses, it is possible that other health care practitioners could benefit from the training.

### Conclusion

Information and communication technology is instrumental in the future of health care practitioners’ education. Indeed, technology is ubiquitous in clinical, professional, and academic settings. Researchers should consider a wide variety of factors, to provide rich, interactive, tailored Web-based e-learning and enhance health care practitioners’ knowledge, skills, and clinical interventions. The optimization of factors related to system quality and technology acceptance could contribute to the way in which care is learned, planned, and provided in health care settings for years to come. Further research is required to improve understanding of health care practitioners’ interactions and technology use in learning, and the impact of Web-based e-learning on patient care. Our results showed that the Web-based e-learning platform for brief MI was feasible and acceptable according to nurses in cardiovascular care. Moreover, the preliminary posttraining results regarding perceived skill and clinical use of brief MI were all more favorable than those observed in the pretraining assessment. MOTIV@CŒUR, which includes role-modeling videos, could introduce nurses to brief MI for the reduction of cardiovascular risk and exert an impact on their skills regarding motivational interventions.

Future research should focus on tailoring Web-based e-learning platforms to health care practitioners’ existing knowledge and experience, to provide individualized paths and fulfill specific learning needs. Further, such training would benefit from the inclusion of additional interactivity measures to enhance the learning experience.

## Appendix

Multimedia Appendix 1 CONSORT-EHEALTH Checklist V 1.6.1.

Multimedia Appendix 2 Screenshots.

## Abbreviations

CCU: coronary care unit
CNP: cardiology nurse practitioner
MI: motivational interviewing
S1: first session of MOTIV@CŒUR
S2: second session of MOTIV@CŒUR
