# Supplementary figures and images for: Evaluation of a Web-Based E-Learning Platform for Brief Motivational Interviewing by Nurses in Cardiovascular Care: A Pilot Study
Source: J Med Internet Res. 2016 Aug 18;18(8):e224. doi: 10.2196/jmir.6298 (PMC5010651; doi:10.2196/jmir.6298)

## Slide 1
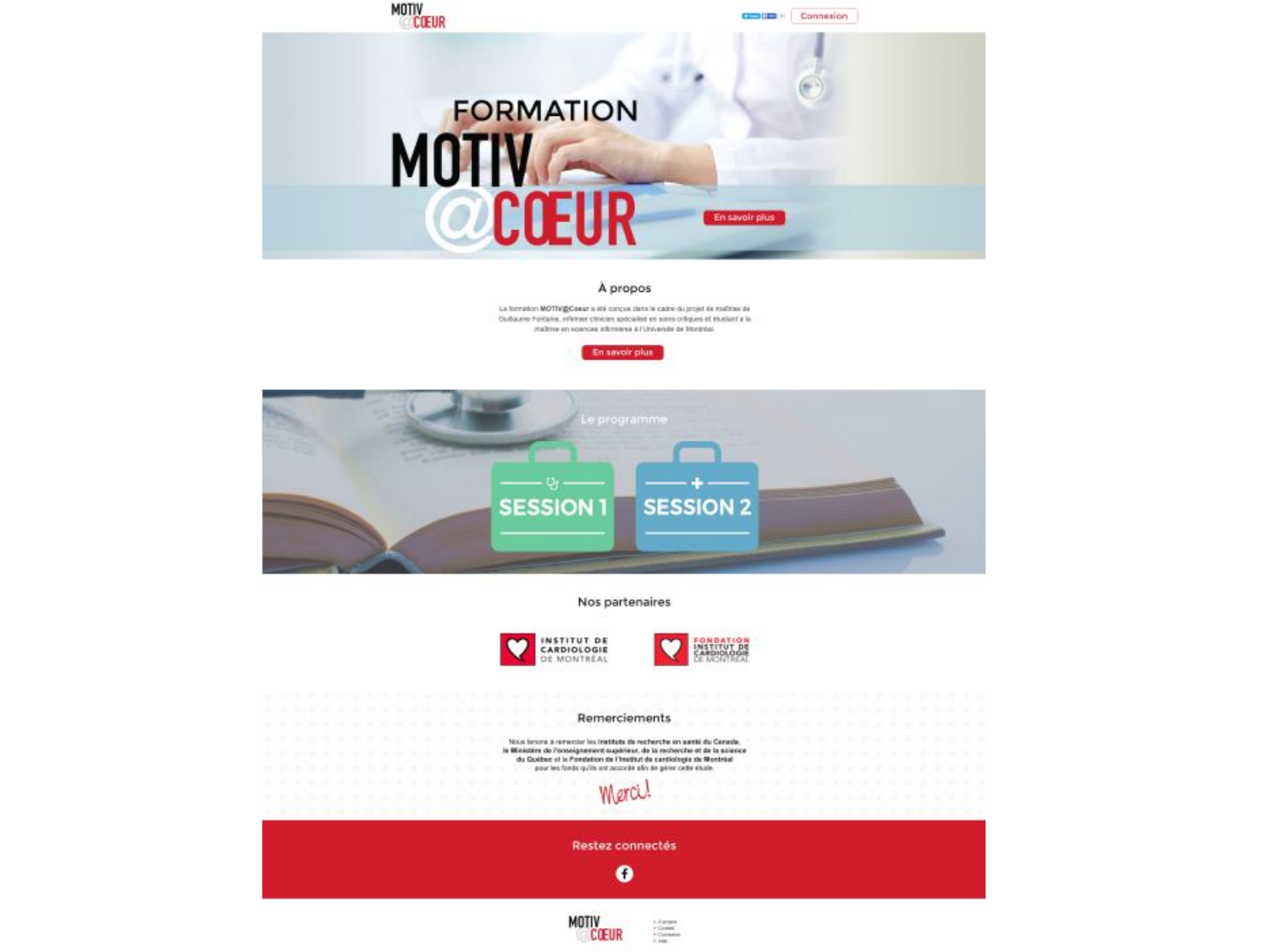

## Slide 2
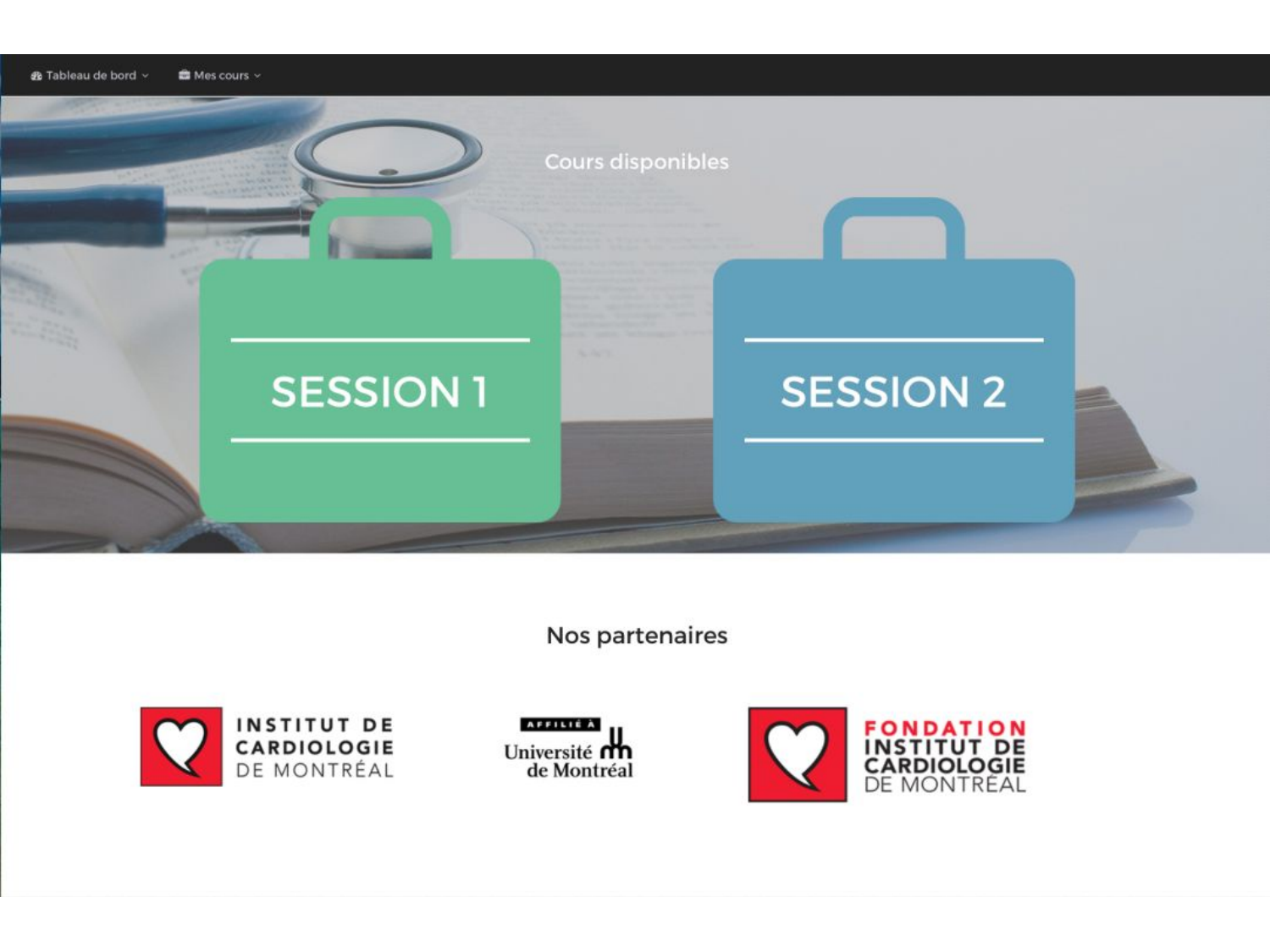

## Slide 3
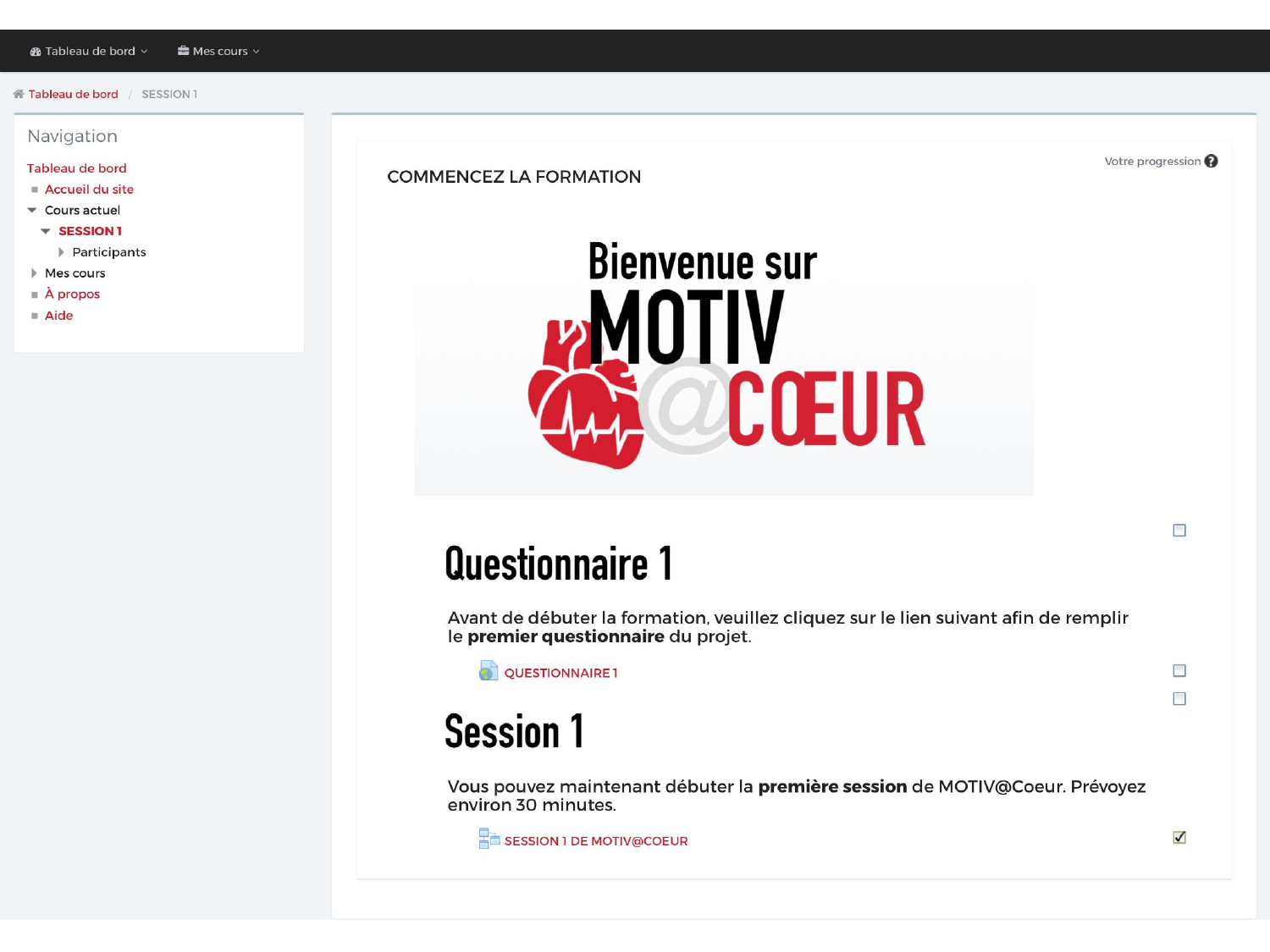

## Slide 4
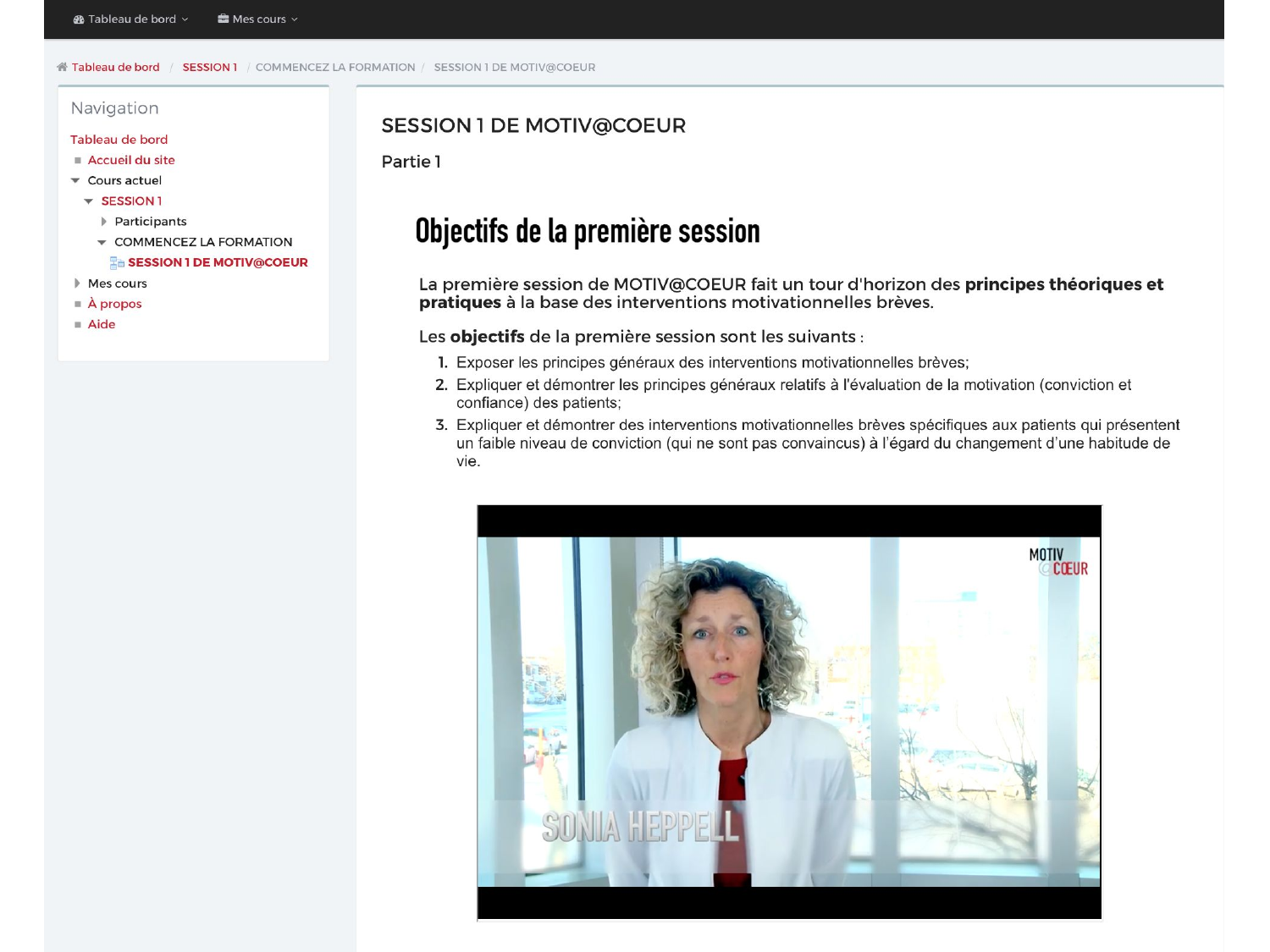

## Slide 5
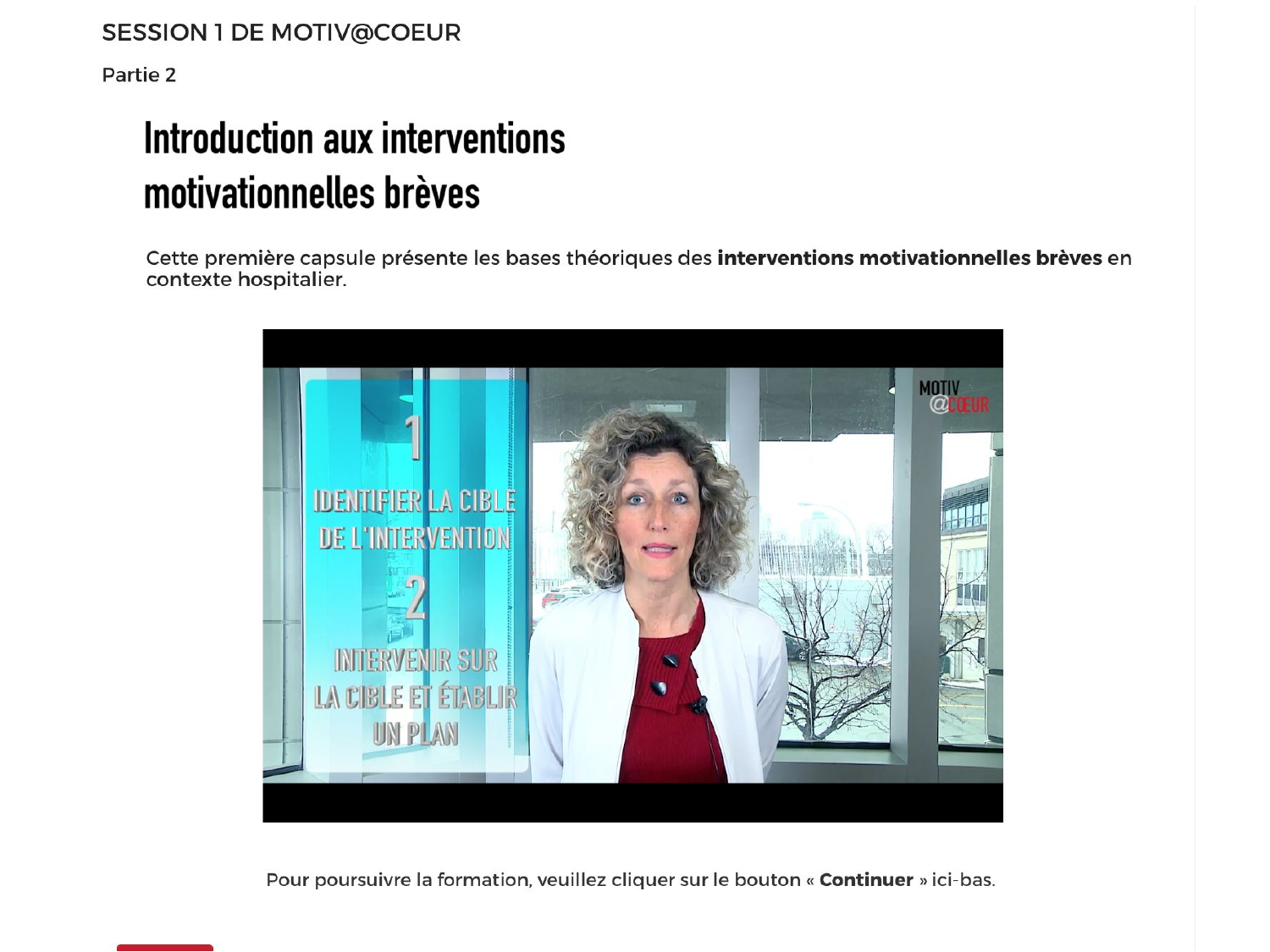

## Slide 6
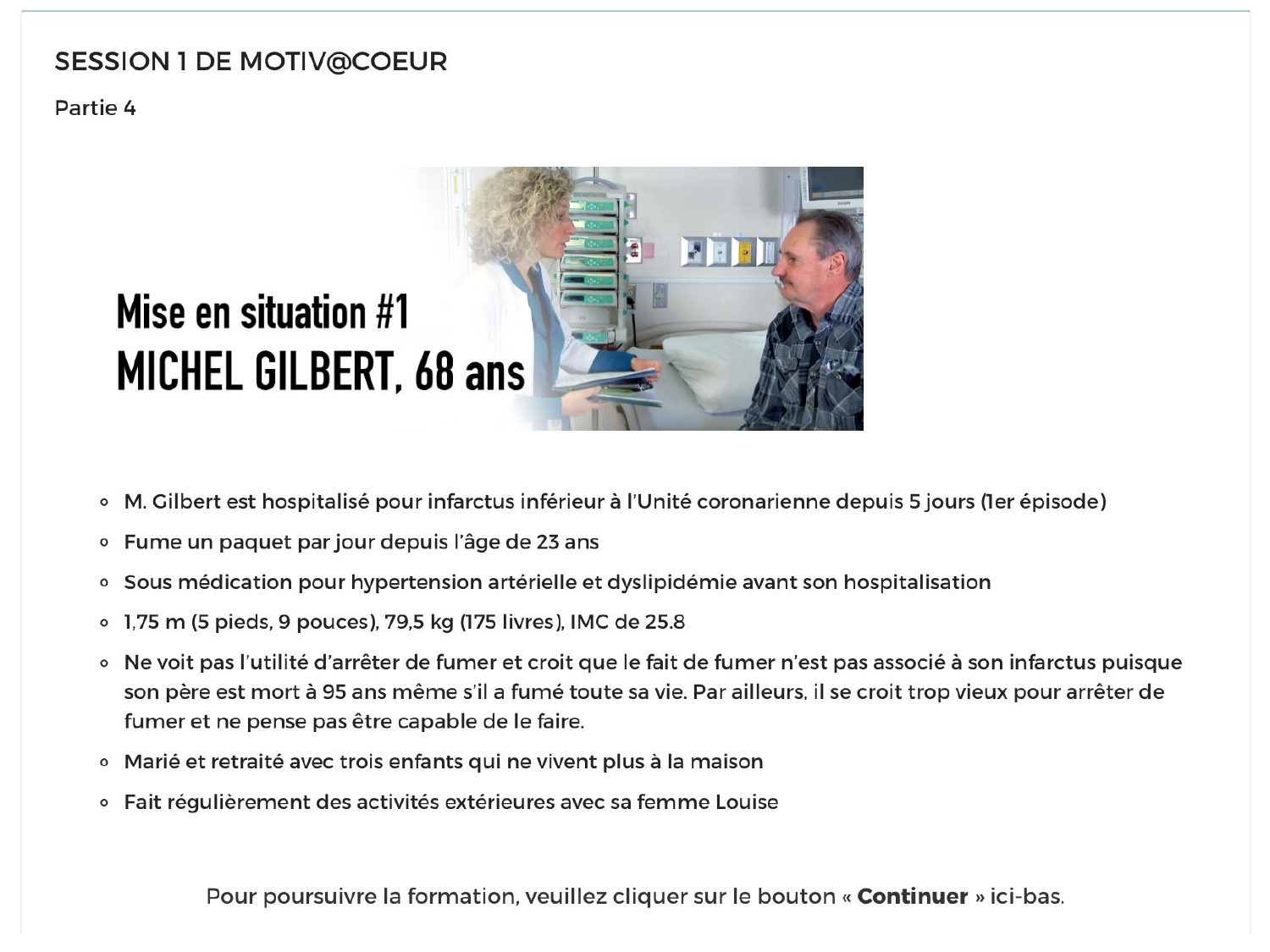

## Slide 7
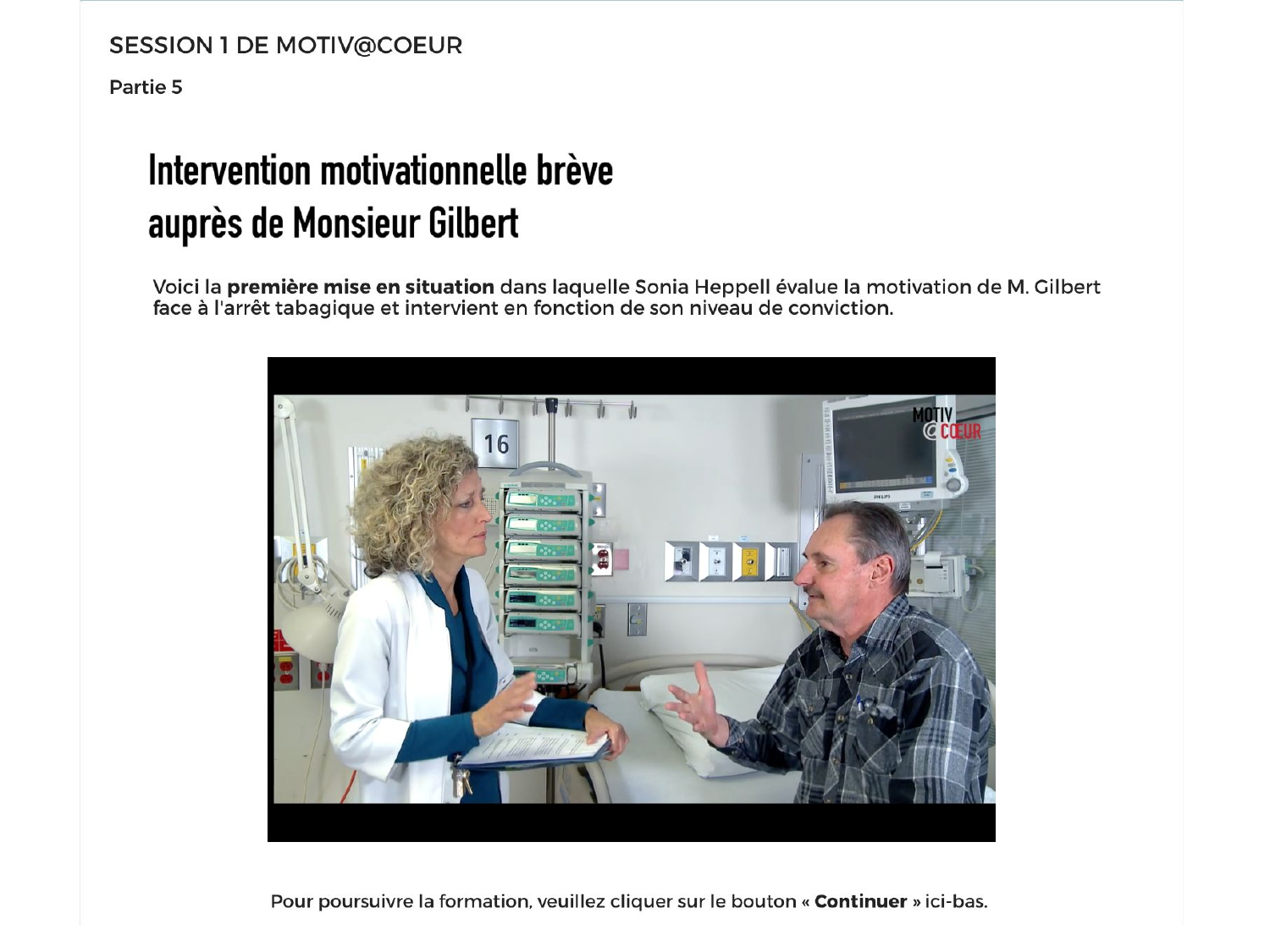

## Slide 8
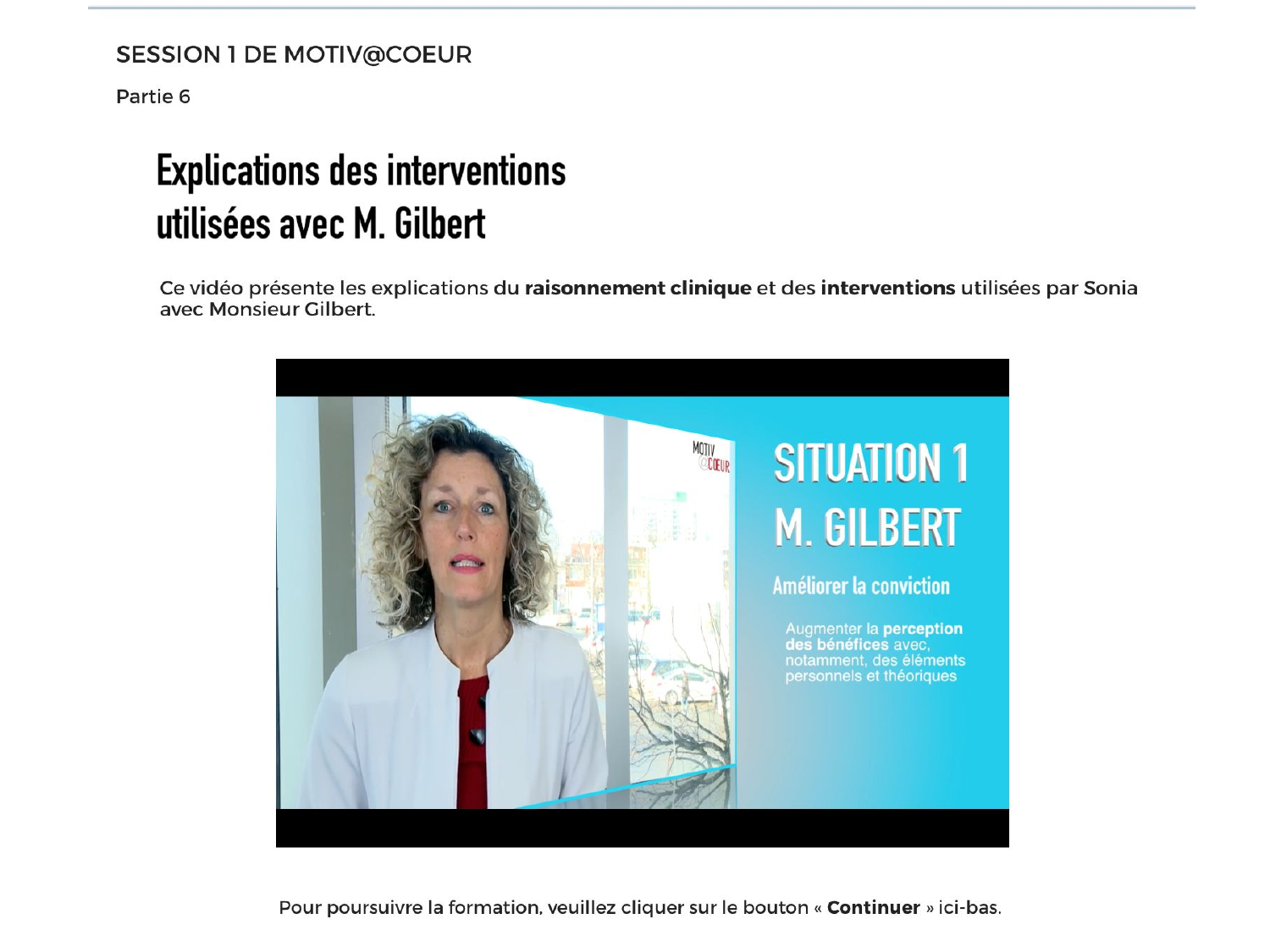

## Slide 9
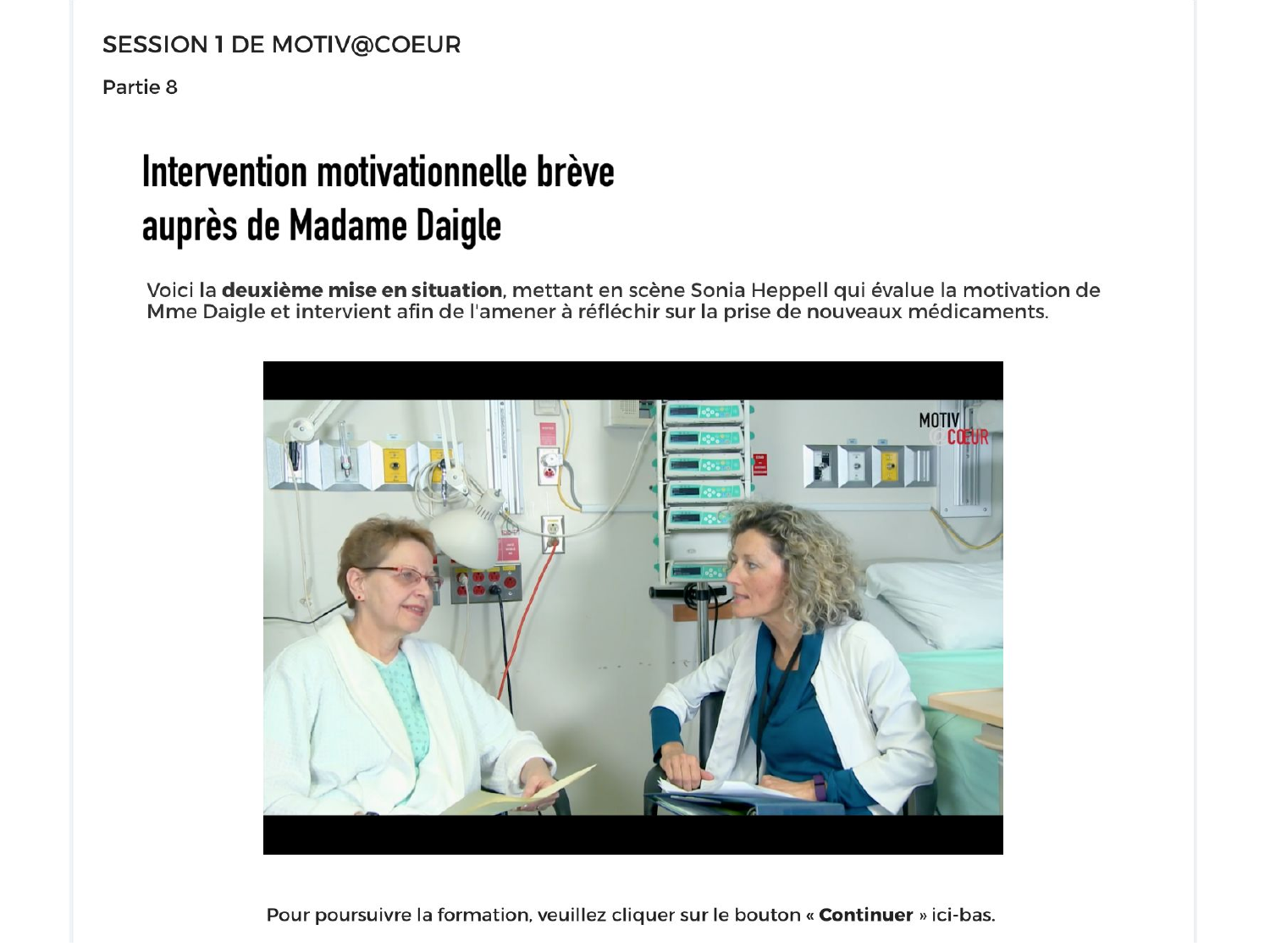

## Slide 10
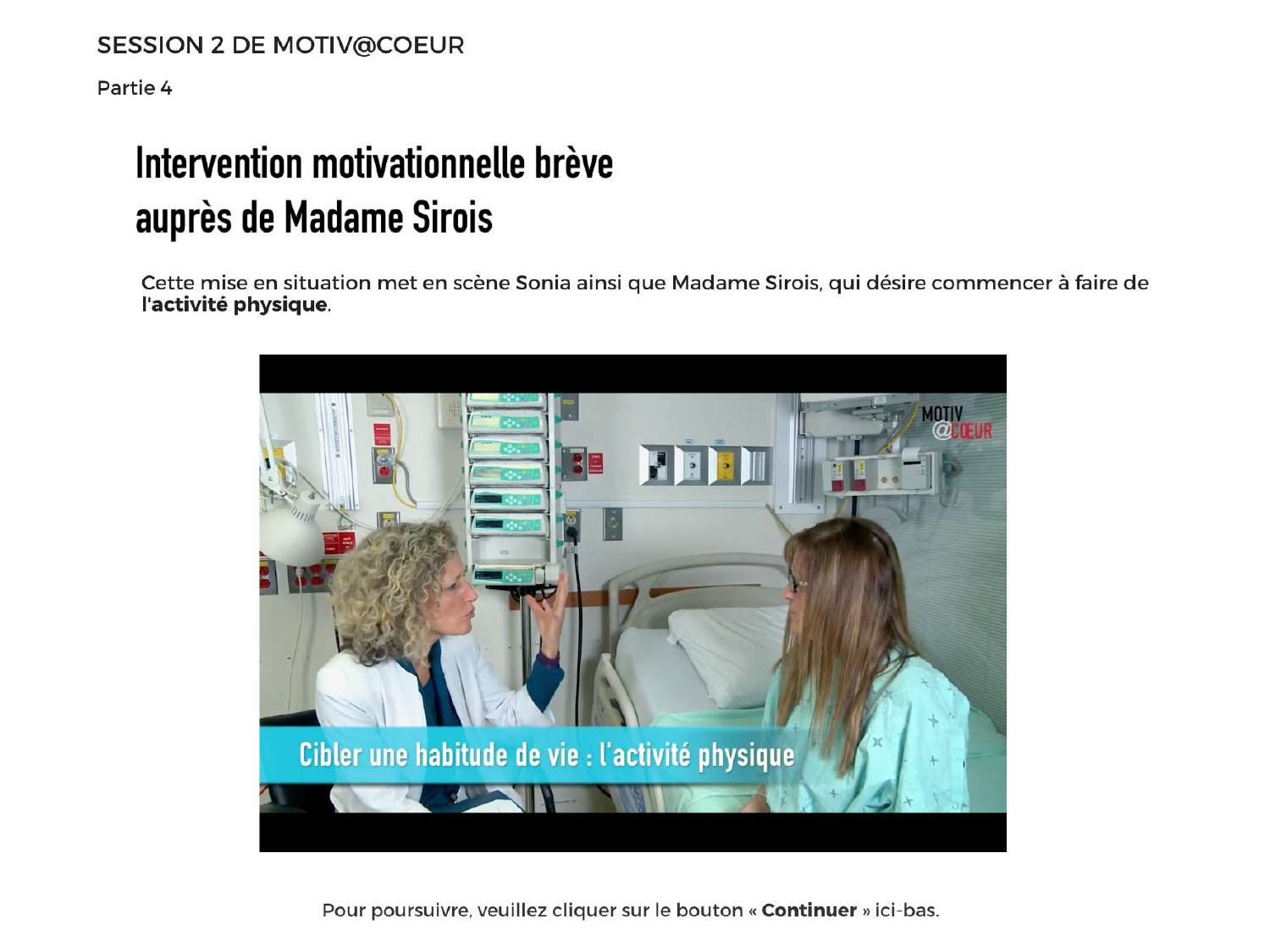

## Slide 11
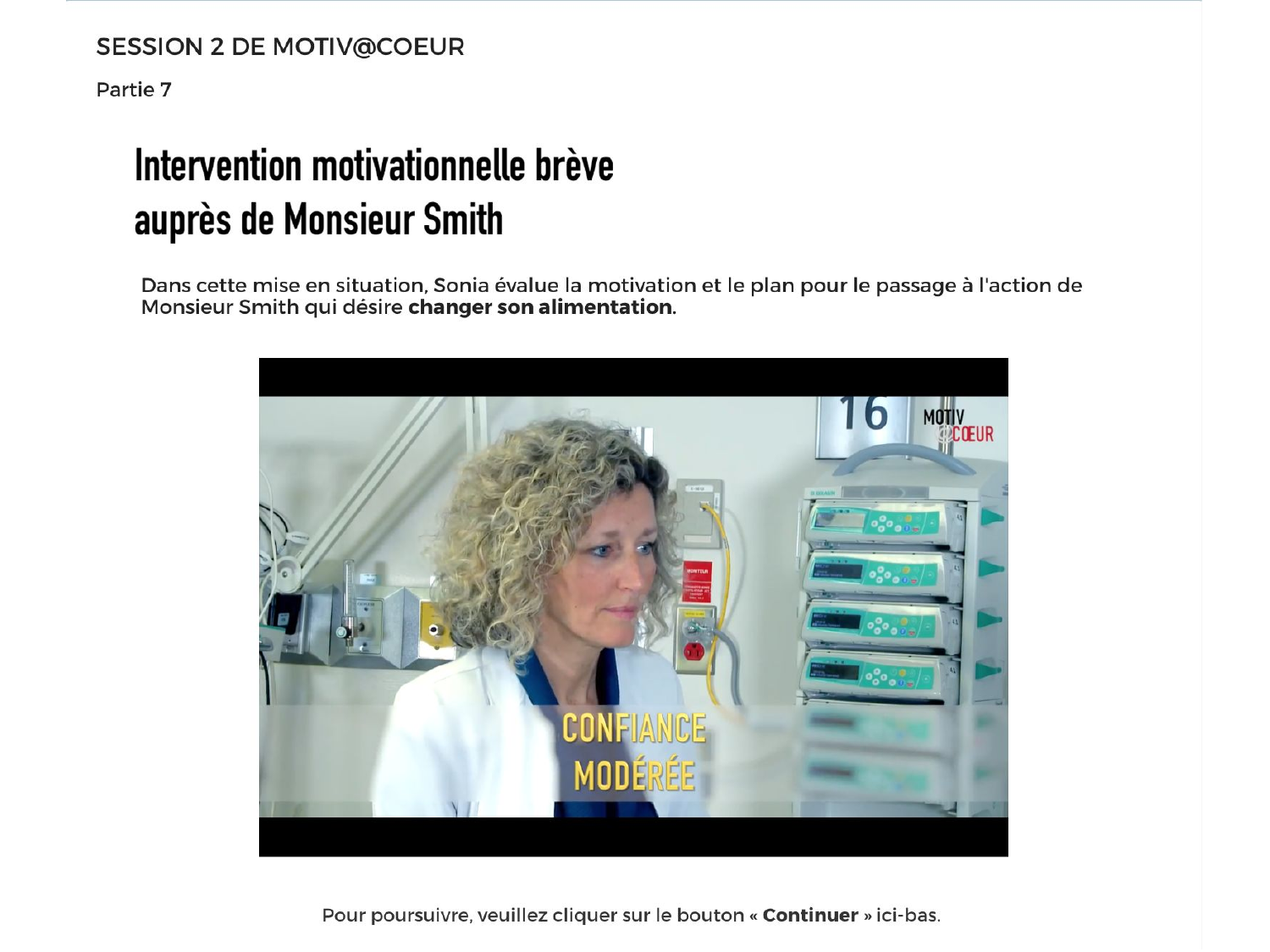

Supplement: Multimedia Appendix 2 [file jmir_v18i8e224_app2.pptx]
